# Fracture Incidence of Kedo-S Square Pediatric Rotary Files: A Prospective Clinical Study

**DOI:** 10.1055/s-0041-1735935

**Published:** 2021-12-04

**Authors:** Lakshimi Lakshmanan, Ganesh Jeevanandan, Prabhadevi C Maganur, Satish Vishwanathaiah

**Affiliations:** 1 Department of Pediatric and Preventive Dentistry – Saveetha Dental College, Saveetha Institute of Medical and Technical Science, Chennai, Tamil Nadu, India; 2 Department of Preventive Dental Sciences, Division of Pedodontics, College of Dentistry, Jazan University, Jazan, Kingdom of Saudi Arabia

**Keywords:** biomechanical preparation, fracture incidence, instrument fracture, Kedo-S Square, rotary file

## Abstract

**Objective** The primary focus of this clinical study was to analyze the probability of occurrence of instrument fracture after root canal preparation of primary molars with the help of Kedo-S Square pediatric rotary file.

**Materials and Methods** Three experienced specialists treated 100 primary maxillary and mandibular molars (335 root canals) using a standardized protocol over 2 months. Biomechanical preparations were carried out using Kedo-S Square file, as per the suggestions given by the manufacturer. Every instrument in Group A helped handle three clinical cases, while for groups B, C and D, they helped in handling 5, 9, and 12 cases, respectively. Making use of an operational microscope, the rotary files, after being pulled out from the canal, were observed. The values were tabulated, and descriptive statistics were performed.

**Results** There were two fractures (2%), of which 1 occurred in group C in the apical 1/3rd of distobuccal canal of maxillary molar, and the other occurred in group D in the apical 1/3rd of mesiobuccal canal of maxillary molar.

**Conclusions** The fracture rate of Kedo-S Square rotary file is quite low. It is primarily in the buccal canals of the maxillary molars and the apical third of the root canal that the instrument has a greater probability of separation.

## Introduction

There is no doubt that pulpectomy is a highly challenging practice in the field of pediatric dentistry and several factors including diagnostic acuity, instruments utilized, and technologies are some crucial factors that contribute to its success.

The endorsement of nickel-titanium (Ni-Ti) rotary instrumentation by the pediatric dentist has expanded two-fold over the most recent years. 1 Although a majority of pediatric dentists utilize rotary instrumentation, there is a critical divergence in the variety, frequency, and method of different instrumentation systems employed.

In the practice of endodontics, intracanal instrument fracture encountered during mechanical preparation of root canals is considered the most objectionable complication. 2 Literature survey reveals that the fracture incidence of rotary instruments is proportionately higher, ranging between 1.3% and 10.0%. 3 However, the higher fracture incidence rate reported in *in vitro* studies varied when used clinically. This likely implies that the outcomes in these cases are being addressed with regard to their clinical pertinence without addressing the true clinical circumstances. 4

Ni-Ti rotary files that are proposed for permanent teeth have been used for canal preparation in primary teeth. Considering the complex morphology of primary teeth roots and its variation from permanent teeth, exclusive pediatric rotary file systems were introduced *de novo* . 5 Continuous upgrades are being made to the design to enable superior and cautious shaping with minimal hindrance to procedural lapse, including canal transportation, ledging, and instrument separation. One such progression in the pediatric rotary file system is the Kedo-S Square file (KEDO Dental, India), a 4th generation file. It is a single-file system designed with a triangular cross-section at the apical region and a tear-drop cross-section at the coronal region. Studies have reported that this file system results in a better quality of obturation in a limited time. 6 Unfortunately, the probability of fracture occurrence linked to the single file system was not available in the literature. Hence, this study was mainly designed with the unilateral aim of analyzing the odds of Kedo-S Square pediatric rotary file fracture in primary molars while performing intracanal procedures and also when working with the instruments in various clinical cases.

## Materials and Methods

This clinical investigation was conducted in a dental institution's Department of Pedodontics and Preventive Dentistry after receiving ethical approval from the Institutional Review Board (SRB/SDC/PEDO-1803/20/01) and registering the trial at clinical trials.gov (CTRI/2021/02/031512). The guidelines of Strengthening the Reporting of Observational Studies in Epidemiology (STROBE checklist) were followed for reporting the results.

Participation was purely voluntary in nature, and as the participants were children, parental assent authorizing the usage of the treatment were obtained.

The study made use of 100 teeth from pediatric volunteers in the age group of 4 to 9 years. Of the 100 teeth, 45 of them were maxillary and 55 were mandibular primary molars. Only those primary molars that had adequate crown structure and whose two-thirds of the root structure was at least intact were included for research, as these were the only ones requiring a single-visit pulpectomy. 5

Those teeth with canal calcifications, sinus tract and pathological resorptions, or those that had been previously root treated, were excluded from the study.

Kedo-S Square rotary file was used for all the instrumentation procedures. Based on computer-generated randomization, and sequentially numbered, opaque and sealed envelope methods, the teeth were randomly assigned to four groups of 25 teeth each. In group A, each of the instruments included was used for three clinical cases, whereas in groups B, C, and D, it was used for 5, 9, and 12 cases, respectively, as the instrument could be used up to 12 times as per the recommendations given by the manufacturer. The complete treatment procedure was carried out by a group of three highly professional and trained pediatric dentists who were well-aware of the system being evaluated. They were also standardized and calibrated to a preendorsed regulation as the one elaborated hereon.

The tooth subjected to study was isolated using a rubber dam only after being subjected to local anesthesia that contained 2% lignocaine in a ratio of 1:200,000 adrenaline (LOX × 2% Adrenaline, Neon Laboratories Ltd., India). While the access cavity was readied using the #6 round bur (Mani, Inc., Japan), the deroofing pulp chamber was done using the nonend cutting burr (Mani, Inc., Japan) such that the researcher is given straight-line access. The canal patency was confirmed with #15 size hand K-file (Mani, Inc. Tochigi, Japan), and the working length was determined with a radiograph with the help of Ingle's method. The Kedo-S Square rotary file was used for biomechanical preparation until the working length, depending on the groups assigned for each. The speed of the endodontic motor (X-Smart Plus electric motor, Dentsply, India Pvt. Ltd., Delhi, India) used was exactly 250 rpm, and the torque measurement stood at 2.2N, both of which met the recommendations given by the manufacturer to the dot. After every single use, each of the rotary files was analyzed for any signs of fracture with the help of a dental operation microscope that had an 8-time magnification effect. During intracanal instrumentation, 17% ethylenediaminetetraacetic acid (EDTA) (Endo Prep RC) was used for lubrication. Intermittent irrigation was carried out with normal saline with 5 mL standard volume. The canals were sufficiently dried with absorbent paper points and blocked with Metapex (Meta Biomed Co. Ltd. Chungbuk, Korea). A complete restoration was established using type II glass ionomer cement from GC, India, and stainless steel crown (3M ESPE).

After the treatment, the clinician recorded a standardized questionnaire with data on probable fracture, the site of occurrence (the coronal, middle, or apical third of root canal), and the usage of any procedures that helped to avoid or retrieve any of the fractured instruments. After tabulation of all the data, the descriptive statistics were performed using SPSS software version 23.0 (SPSS Inc., Chicago, IL, USA).

## Results

Among the 100 participants included in the study, 43 of them belonged to the male gender and 57 of them belonged to the female gender. All the participants belonged to the age group of 4 to 9 years, while the average age was 6.4 ± 1.2 years. Table 1 provided below gives a clear insight into the details of the study sample, such as the type of tooth and the root canal statistics in each of the groups. Table 2 gives details regarding the total count of fractured instruments, considering the teeth and root canals present in each group. The fractured instruments were bypassed.

**Table 1:** Distribution of study samples in terms of teeth type and root canals

Groups	No. of mandibular teeth	No. of maxillary teeth	No. of mandibular canals	No. of maxillary canals
Group A	15	13	58	39
Group B	13	10	50	30
Group C	12	11	42	33
Group D	15	11	50	33
Total	55	45	200	135
	100 teeth		335 canals	

**Table 2:** Distribution of fracture incidence, considering the tooth type and location

Groups	No. of fractured instrument	Canal/tooth	Location (root)
Group A	0	−	−
Group B	0	−	−
Group C	1	Distobuccal canal of maxillary 2nd molar	Apical 3rd
Group D	1	Mesiobuccal canal of maxillary 2nd molar	Apical 3rd

## Discussion

The establishment and advancement of Ni-Ti rotary instruments are assuredly on an upswing in the field of endodontics. Each file system has unique qualities relating to the benefits and hindrances, and specific standards for its use are also to be followed. The Kedo-S Square rotary file system was launched with the promise of lesser root dentin preparation offering decreased rate of primary root resorption. This file system with variably variable taper abrades the dentin, thereby removing a thin layer of dentin from the entire perimeter of the root canal and maintaining dentin integrity for successful three-dimensional obturation, unlike other rotary files with large taper removing excessive dentin, resulting in weakening of roots. 7

In comparison to a laboratory investigation, this prospective clinical trial delivers more efficient and stronger proof, but only if each of the operators follows the usual protocol standards. However, a laboratory study cannot guarantee a clinical scenario each time. 8

Every root canal preparation performed in this study met all the recommendations given by the manufacturer besides adhering to the standardized procedure suggested. Although it is usually preferable to go for single-use to reduce the risk of rotary instrument fracture, the higher costs associated with rotary files persuade operators to reuse them frequently. Various determinants influence the safe reuse of rotary files, such as the total number of times the file is reused, preparation technique, glide path preparation, adequate orifice enlargement before rotary instrumentation, enlargement of the root canal using conventional hand files, and the utilization of adequate irrigants or lubrication along with the rotary files. 9

In the current study, the #15 size hand files were the only ones used to create the glide path without using any other instruments. The best possible way to minimize instrument fracture is to prepare a manual guide path before performing rotary instrumentation. 10 Kedo-S Square file system was operated in brushing motion as per recommendation. Such brief use of the instrument and the light apical pressure is beneficial in reducing the risk of rotary instrument fracture in contrast to the continuous pecking motion. Further, EDTA was utilized for smear layer removal and lubrication, which proved to reduce the probability of instrument fracture. 11 The crown down technique employed is reported to minimize coronal interference, as a result of which both torque load and any errors during the procedure are eliminated as much as possible. 9

A strikingly arguable issue in endodontics is how many times an endodontic file can be used safely for root canal preparation. Several studies have reported that it is conceivable to reuse the instruments. 8 10 12 13 Yared et al in a simulated clinical study found that up to four molar teeth could be prepared using the same rotary instrument without the fear of instrument fracture. 14 Parashos et al reported that the incidence of fracture of rotary instruments may indeed be lower than that for stainless steel manual files, and the reasons for fracture of rotary instruments are intricate and multifactorial. 15 It is also seen that how the operator handles the instruments and his/her discretion on the maximum number of times up to which an instrument could be used are major pointers that could affect the rate of instrument fracture, as per various studies. 16

Literature shows there is no relation found between the total number of times an instrument being used and the rate of instrument fracture. 15

Research shows that factors such as the properties of the instrument used, root canal morphology, and the operator's proficiency affect the maximum number of times a rotary file can be used. These inferences show that there are no valid proofs available in the literature supporting the one-time use of rotary files to reduce fracture rate incidence, and it can only be a suggestive opinion that could be considered. 15 17

Although the Kedo-S Square rotary instrument could be thrown away only after being used 12 times, as per the recommendations by the manufacturer, it is observed that the wear and tear subjected to the instrument varies greatly, depending on whether it is used for a tooth with a single canal or for a tooth having numerous canals. The study here was taken up to shed light on this dilemma.

After every use for root canal preparation, the rotary files were observed for any chances of the fracture using a dental operating microscope that had 8-times magnifying power. This 8x magnification was supported by Bueno et al, as they considered this level to be most commonly used by operators. 13

In the present study, the incidence of instrument fracture was found to be 2% and the instrument fracture occurred in apical 1/3rd of root canals. This was in accordance with the survey conducted among various endodontists who reported that instrument fracture is 33.5 times likelier to occur in the apical 1/3rd where canals commonly curve and have their least diameter when compared to the coronal 1/3rd of root canals. 18 Marked root curvatures increase the possibility of instrument fracture as there are higher possibilities of higher cyclic fatigue. 19

The advantages of heat treatment that assist in enhancing flexibility, protect against cyclic fatigue, and provide cutting efficiency are all assimilated by Kedo-S Square file. The thermal regimen is further responsible for the controlled memory effect on the files. 6

An instrument's resistance to fight against fracture, even under the pressure of flexural and torsional load, varies based on its cross-sectional area design and file design. In general, those instruments bearing vast diameters are more easily susceptible to flexural fatigue in comparison to those with a smaller diameter, and such instruments are also subjected to greater internal stress accumulation. 20

Any instrument that is designed to minimize the contact area that exists between the instrument and the root dentinal walls is sure to decrease the vertical load on it. 21 This could explain the low fracture incidence with Kedo-S Square file system, as the diameter of the file is 0.28 mm with a dual cross-sectional area (triangular cross-section at apical region and teardrop cross-section at coronal region) and has variably variable taper.

The possibilities of managing any fractured instrument are dependent on several factors, while removing such instruments is primarily dependent on factors such as the tooth's etiology, the maximum degree of canal curvature, and the exact site of the fragment. 22 Every fractured instrument in this study was bypassed and patients were kept under periodic follow-up.

Some of the limitations of this present study include the heterogeneity of the sample, the most important of which are the degree of root curvature, the total number of root canals, and the absence of other file systems that could be used for comparison. To reconfirm the current study's results concerning limited fracture incidence, an investigation probing into teeth samples that consist of varied canal configurations, making use of altered treatment protocols, and involving operators who hold various work experiences is necessary.

## Conclusion

The present study concludes that the rate of fracture of Kedo-S Square rotary file is lower, considering the study criteria. There are higher chances that the instrument might separate in the buccal canals of the maxillary molars and the apical third of the root canals.
